# PSPC1 is a new contextual determinant of aberrant subcellular translocation of oncogenes in tumor progression

**DOI:** 10.1186/s12929-021-00753-3

**Published:** 2021-08-02

**Authors:** Yaw-Dong Lang, Yuh-Shan Jou

**Affiliations:** Institute of Biomedical Sciences, Academia Sinica, 11529 Taipei, Taiwan

**Keywords:** PSPC1, Nucleocytoplasmic shuttling, Oncogenic switch, Selective inhibitor of nucleocytoplasmic shuttling, TGIF1, NPM, Mortalin and EBP50

## Abstract

Dysregulation of nucleocytoplasmic shuttling is commonly observed in cancers and emerging as a cancer hallmark for the development of anticancer therapeutic strategies. Despite its severe adverse effects, selinexor, a selective first-in-class inhibitor of the common nuclear export receptor XPO1, was developed to target nucleocytoplasmic protein shuttling and received accelerated FDA approval in 2019 in combination with dexamethasone as a fifth-line therapeutic option for adults with relapsed refractory multiple myeloma (RRMM). To explore innovative targets in nucleocytoplasmic shuttling, we propose that the aberrant contextual determinants of nucleocytoplasmic shuttling, such as PSPC1 (Paraspeckle component 1), TGIF1 (TGF-β Induced Factor Homeobox 1), NPM1 (Nucleophosmin), Mortalin and EBP50, that modulate shuttling (or cargo) proteins with opposite tumorigenic functions in different subcellular locations could be theranostic targets for developing anticancer strategies. For instance, PSPC1 was recently shown to be the contextual determinant of the TGF-β prometastatic switch and PTK6/β-catenin reciprocal oncogenic nucleocytoplasmic shuttling during hepatocellular carcinoma (HCC) progression. The innovative nucleocytoplasmic shuttling inhibitor PSPC1 C-terminal 131 polypeptide (PSPC1-CT131), which was developed to target both the shuttling determinant PSPC1 and the shuttling protein PTK6, maintained their tumor-suppressive characteristics and exhibited synergistic effects on tumor suppression in HCC cells and mouse models. In summary, targeting the contextual determinants of nucleocytoplasmic shuttling with cargo proteins having opposite tumorigenic functions in different subcellular locations could be an innovative strategy for developing new therapeutic biomarkers and agents to improve cancer therapy.

## Introduction

Nucleocytoplasmic shuttling is the dynamic physiological movement of macromolecules, including proteins and RNAs, into the proper cellular compartments for performing their designated biological functions and maintaining cellular homeostasis [1]. Although nucleocytoplasmic protein shuttling has been observed for decades, the detailed molecular mechanisms and their translational applications have only recently been explored. Indeed, the protein transportation process is complex and tightly coordinated in both directions across the nuclear envelope, and it includes sequential protein interaction steps to (a) recognize the protein import or export signal by an import or export receptor, (b) dock the transporting protein with the import/export receptor to nuclear pore complexes (NPCs), (c) translocate the shuttling protein across the nuclear pore, (d) release the transported protein, and then (e) recycle the transport factors in the shuttling machinery to facilitate protein shuttling [2].

In eukaryotic cells, the contextual determinant of nucleocytoplasmic shuttling is not only responsible for propagating signaling by transducing internal and external stimuli such as signals from growth factors (e.g., TGF-β), cytokines (e.g., IL-6), or even stresses to activate signal transducers such as Smads, β-catenin, ERKs, STAT3, p53, and NF-κB by posttranslational modification, but also these factors then enter the nucleus to interact with spatiotemporal cofactors in the designated cellular environment for activation of downstream signaling and responses with proper cellular functions [3]. Generally, canonical shuttling proteins contain a nuclear localization signal (NLS) domain for importin-mediated protein import into the nucleus or a nuclear export signal (NES) domain for the nuclear export receptor CRM1 (chromosome region maintenance 1, also called exportin 1, XPO1) for export into the cytoplasm [4]. A well-studied example is that DNA damage can induce the phosphorylation of the cytoplasmic tumor suppressor protein p53 by kinases, including ataxia telangiectasia mutated (ATM) and ATM-Rad3-related (ATR), followed by interaction of the p53 NLS-like domain with importins to dock on NPCs for nuclear import [5]. After p53 acetylation by p300/CREB-binding protein (CBP) to enhance the p53 DNA-binding affinity, p53 accumulated in the nucleus in response to DNA damage can mediate cell cycle arrest, apoptosis and senescence [6]. Then, ubiquitylation (Ub) of p53 mediated by murine double minute 2 (MDM2), an E3 ligase, transports p53 via its NES-like motif to CRM1, leading to the export of nuclear p53 into the cytoplasm for ubiquitin-dependent proteasomal degradation. The elegant and dynamic modulation of p53 nucleocytoplasmic shuttling via the MDM2 interaction helps to maintain a low level of p53 in normal cells and to deal with damage-induced stress by increasing the p53 level to maintain cell homeostasis.

Dynamic nucleocytoplasmic shuttling is a crucial mechanism to keep proteins in the appropriate subcellular localization and to allow them to perform proper functions in a given physiological context. Therefore, aberrancies in the proteins involved in nucleocytoplasmic protein shuttling can lead to incorrect subcellular localization of proteins associated with human diseases. Some review articles have provided a comprehensive summary of potential aberrancies in the protein trafficking machinery, including mutations and/or aberrant expression of protein shuttling signals, transporters in the shuttling machinery, and posttranslational modifications of cargo and signal transducers causing alterations in protein interaction networks and aggregation of misfolded proteins leading to mislocalization of cargo proteins associated with human diseases such as cancer, neurodegenerative diseases, osteoporosis and anemia [4, 7, 8].

With the body of information on the mechanisms of nucleocytoplasmic shuttling generated over the past few decades, aberrant subcellular localization of oncogenes and tumor suppressor genes has been found to occur frequently in cancers and promote tumorigenesis, metastasis and drug resistance in divergent cancer types [9, 10]. Therefore, aberrant nucleocytoplasmic shuttling of proteins and RNAs has emerged as a hallmark of cancer with great potential to serve as a theranostic target for cancer therapy. Indeed, it has been reported that XPO1 exports at least 221 NES-containing proteins plus a subset of nuclear RNAs from the nucleus to the cytoplasm. Selective inhibitors of nuclear transport (SINEs) are novel inhibitors of XPO1 that target the protein-interacting residue Cys528 in the NES-binding pocket of XPO1 as orally bioavailable inhibitors including KPT-185, KPT-251, KPT-276, selinexor, eltanexor and verdinexor [11–15]. Selinexor, a first-in-class nuclear export inhibitor targeting canonical nucleocytoplasmic shuttling, causes the accumulation of tumor suppressor proteins accompanied by a reduction in oncoproteins. Selinexor was approved by U.S. Food and Drug Administration (FDA) in 2019 in combination with dexamethasone as a fifth-line therapeutic option for adult patients with relapsed refractory multiple myeloma (RRMM) [16]. However, the use of SINEs in cancer therapy remains challenging because of their low therapeutic efficacy against solid tumors in phase I and II clinical trials and their severe adverse effects of low platelet counts and low blood sodium levels that lead to thrombocytopenia, neutropenia, fatigue, and nausea [17].

Owing to advances in the molecular pathological mechanisms of diseases, different nucleocytoplasmic shuttling (or cargo) proteins have been uncovered, including categories of transcription factors/cofactors (e.g., STAT3, p53, etc.; reviewed in [18, 19]), RNA-binding proteins (e.g., HuR and hnRNPs; reviewed in [20]), hormone and growth factor receptors (e.g., the androgen receptor and EGFR; reviewed in [21, 22]), translation initiation factors (e.g., eIF4E; reviewed in [23]) and kinases (e.g., protein-tyrosine kinase 6, PTK6; also called breast tumor kinase, Brk) [24]. Instead of relying on conventional NLS- and NES-like domains, many of the cargo proteins have been shown to dynamically shuttle between the nucleus and cytoplasm in NLS and NES domain-independent manners (e.g., β-catenin) via interaction with other chaperone or interacting proteins with unknown mechanisms [24, 25]. During both canonical and noncanonical nucleocytoplasmic shuttling, cargo proteins interact with and are sequestered by the “contextual determinants” of nucleocytoplasmic shuttling in a specific subcellular localization in a cell context-dependent manner to perform their designated pathophysiological function. The strategy of developing innovative inhibitors by targeting the contextual determinants of nucleocytoplasmic shuttling might confer some advantages over the conventional design of SINEs targeting common machinery proteins, such as XPO1, in nucleocytoplasmic shuttling. First, contextual determinants of nucleocytoplasmic shuttling are commonly induced under aberrant stresses (e.g., somatic mutations) in a cell content-dependent manner to trigger pathological events. Second, selective targeting of both interacting contextual determinants and cargo proteins might maintain their tumor-suppressive activities and exhibit synergistic tumor-suppressive effects. Finally, specific targeting of a selected contextual determinant of aberrant nucleocytoplasmic shuttling might result in low toxicity to cancer patients, based on clinical experience with targeted therapies in cancers.

PSPC1 (paraspeckle component 1) was recently identified as a contextual determinant of tumor progression in multiple cancer types involving oncogenic reprogramming to switch proapoptotic TGF-β to prometastatic TGF-β via hijacking of Smad2/3 targeting [26]. Moreover, PSPC1, as a substrate of nuclear PTK6, is the contextual determinant of PTK6 nucleocytoplasmic shuttling and modulates the switch of tumor-suppressive PTK6 in the nucleus of normal or premalignant cells to oncogenic PTK6 in the cytoplasm of malignant cancer cells [27]. An innovative dual inhibitor derived from a unique C-terminal polypeptide composed of 131 amino acids of PSPC1 targeting both oncogenic PSPC1 and PTK6 nucleocytoplasmic shuttling was shown to exhibit synergistic tumor-suppressive effects in HCC cell lines and mouse models [28]. These observations elucidated that agents targeting aberrant oncogenic contextual determinants and nucleocytoplasmic shuttling proteins might be a new class of anticancer theranostic targets with the anticancer feature of tissue specificity for reducing cytotoxicity and producing synergistic effects on tumor suppression via combined targeting of shuttling determinant and cargo proteins.

**Fig. 1:**
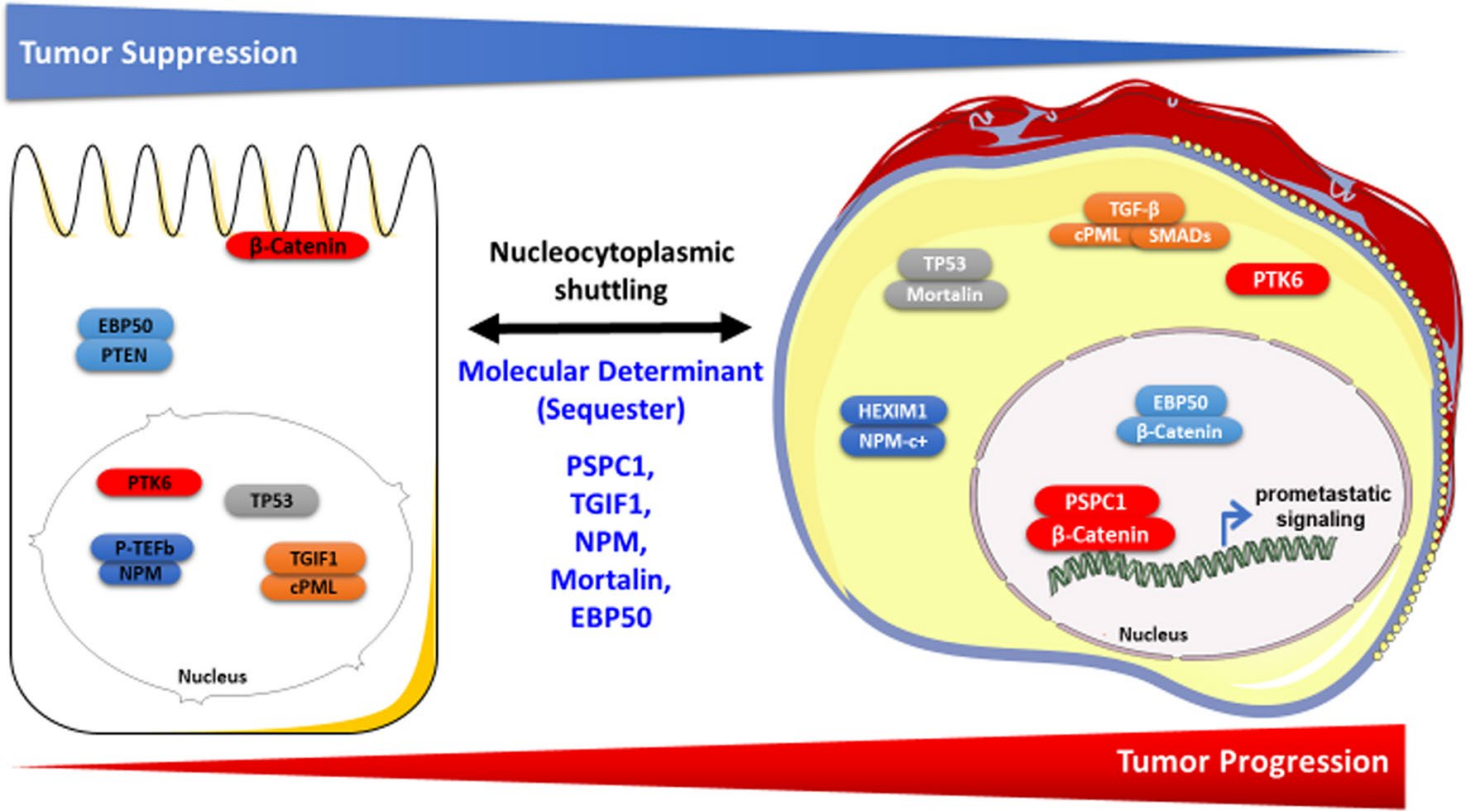
Phenotypic characteristics and contextual determinants of nucleocytoplasmic shuttling in tumor progression. Schematic representation of subcellular localizations of the contextual determinants and nucleocytoplasmic shuttling proteins involved in tumor progression. The left side of the figure shows normal or benign cells with low tumorigenic potential. The right side of the figure shows malignant cancer cells. The contextual determinant and the nucleocytoplasmic shuttling protein involved in the same subcellular translocation event are highlighted in the same color

In this review article, we discuss 5 aberrant contextual determinants of nucleocytoplasmic shuttling—PSPC1, TGIF1, NPM, Mortalin and EBP50—meeting these criteria and note their detailed mechanisms of action as oncogenic nucleocytoplasmic shuttling switches, potential use as biomarkers for stratification of cancer patients, and possibilities as putative therapeutic agents to potentially improve cancer therapy (Fig. 1; Table 1).

**Table 1:** List of contextual determinants of nucleocytoplasmic shuttling with determination of opposite cancerous functions of cargos

Molecular determinant	Shuttling protein	Nuclear function of shuttling protein	Mechanisms of nuclear shuttling protein	Cytoplasmic function of shuttling protein	Mechanisms of cytoplasmic shuttling protein	Potential inhibitor	Biomarker
PSPC1 [27]	PTK6	PTK6 is a tumor suppressor	PTK6 is phosphorylated and trapped by PSPC1	PTK6 is an oncogene	PTK6 interacts with proteins in oncogene networks	PSPC1-CT131	Decreased PSPC1-pY523 expression predicts HCC tumor progression
TGIF1 [29, 30]	PML	PML is a tumor suppressor	TGIF1 sequesters cPML and acts as a negative TGF-β signal regulator	Cytoplasmic PML (cPML) is an oncogene	cPML is exported into the cytoplasm to interact with Smads for activating TGF-β pathway	Arsenic trioxide (ATO) or All-trans-retinoic acid (ATRA)	PML–RARα fusion oncogene in acute promyelocytic leukemia (APL)
NPM [31, 32]	HEXIM1	HEXIM1 is a tumor suppressor	P-TEFb sequesters HEXIM1, resulting in the inactivation of P-TEFb-mediated inhibition of RNA polymerase II transcription	Cytoplasmic NPM mutant (NPMc+) is an oncogene	NPMc + associates with and sequesters HEXIM1, leading to higher RNA polymerase II transcription	Cytotoxic peptide of the basic region (BR) of HEXIM1	NPMc + is the signature for acute myeloid leukemia (AML)
Mortalin [33–35]	P53	P53 is a tumor suppressor	P53 acts as a suppressor to participate in DNA repair, apoptosis, and cell cycle progression	P53 accumulation and retention in the cytoplasm lead to drug resistance and oncogenic features	Cytoplasmic p53 is sequestered by mortalin due to p53 excessive nuclear export, low cytoplasmic degradation, and retention by the cytoskeleton.	P53 peptide (323–337 amino acids), MKT-077, shRNAs of mortalin	Cytoplasmic p53 is associated with poor chemotherapy, metastasis, and poor patient survival
EBP50 [36, 37]	EBP50, β-catenin	EBP50 is an oncogene	EBP50/β-catenin enters the nucleus to stabilize β-catenin/TCF-1 to activate aberrant Wnt signaling	EBP50 is a tumor suppressor	EBP50 interacts with the PTEN tumor suppressor to attenuate PDGF receptor signaling	siEBP50	Nuclear expression of EBP50 is associated with poor survival

## PSPC1 is the contextual determinant of the reciprocal nucleocytoplasmic shuttling of oncogenic PTK6 and β-catenin

PSPC1 (Paraspeckle component 1) is a nuclear protein located at punctate subnuclear structures neighboring splicing speckles, called paraspeckles, that are mainly composed of three Drosophila behavior/human splicing (DBHS) proteins—PSPC1; splicing factor proline- and glutamine-rich (SFPQ), also called PSF; and non-POU-domain-containing octamer-binding protein (NONO), also called p54nrb—and a long noncoding RNA called NEAT1 (Nuclear enriched abundant transcript 1) [38]. PSPC1 is a putative transcription factor/cofactor and is known as an androgen receptor coactivator in differentiating Sertoli cell nuclei [39], participates in the DNA damage response [40], recruits TET2 to ERVL-associated genes for their transcriptional repression via histone deacetylases and posttranscriptional destabilization of RNAs through 5hmC modification [41], promotes the differentiation-dependent nuclear export of adipocyte RNAs [42], potentiates IGF1R expression and augments cell motility [43], and acts as a contextual master driver of prometastatic reprogramming of transforming growth factor β1 (TGF-β1) signaling toward a prometastatic phenotype associated with activating epithelial-to-mesenchymal transition (EMT), stemness and tumor metastasis in multiple cancer types [26, 44].

To better understand the roles of PSPC1 in tumor progression and develop therapeutic strategies to halt tumor metastasis, we also conducted integrated proteomics and transcriptomic analyses and found that PSPC1 is a contextual determinant of tumor progression to potentiate the reciprocal oncogenic switches of cytoplasmic protein-tyrosine kinase 6 (PTK6) and nuclear β-catenin subcellular translocation to augment Wnt3a autocrine signaling in tumor progression [27]. PTK6 (or Brk) belongs to the nonreceptor tyrosine kinase FRK/PTK6 family and is composed of Src homology 3 (SH3), Src homology 2 (SH2) and kinase (SH1) domains; it is known to be upregulated in multiple cancer types and normal epithelial cells [45]. It is well noted that PTK6 acts as a tumor suppressor localized in the nucleus through phosphorylation of some RNA-binding proteins, such as Sam68 and the splicing factor PSF, to promote the cytoplasmic relocalization of RNA-binding proteins and to impair their binding to polypyrimidine RNAs, leading to cell cycle arrest [24]. In differentiated prostate cancer cells, nuclear localization of PTK6 may provide a weak tumorigenic indicator for prostate tumor progression [46]. In contrast, PTK6 has been shown to be overexpressed in multiple cancer types as an oncogene and to be localized in the cytoplasm and at the cell membrane [47]. Cytoplasmic PTK6 can interact with over 30 identified substrates within the cytosol and participate in facilitating oncogenic functions of cancer cells to enhance their migration and invasion [47, 48]. PSPC1 is the first protein substrate to be identified as a contextual determinant of PTK6 subcellular localization accompanied by a switch in the oncogenic function of PTK6.

**Fig. 2:**
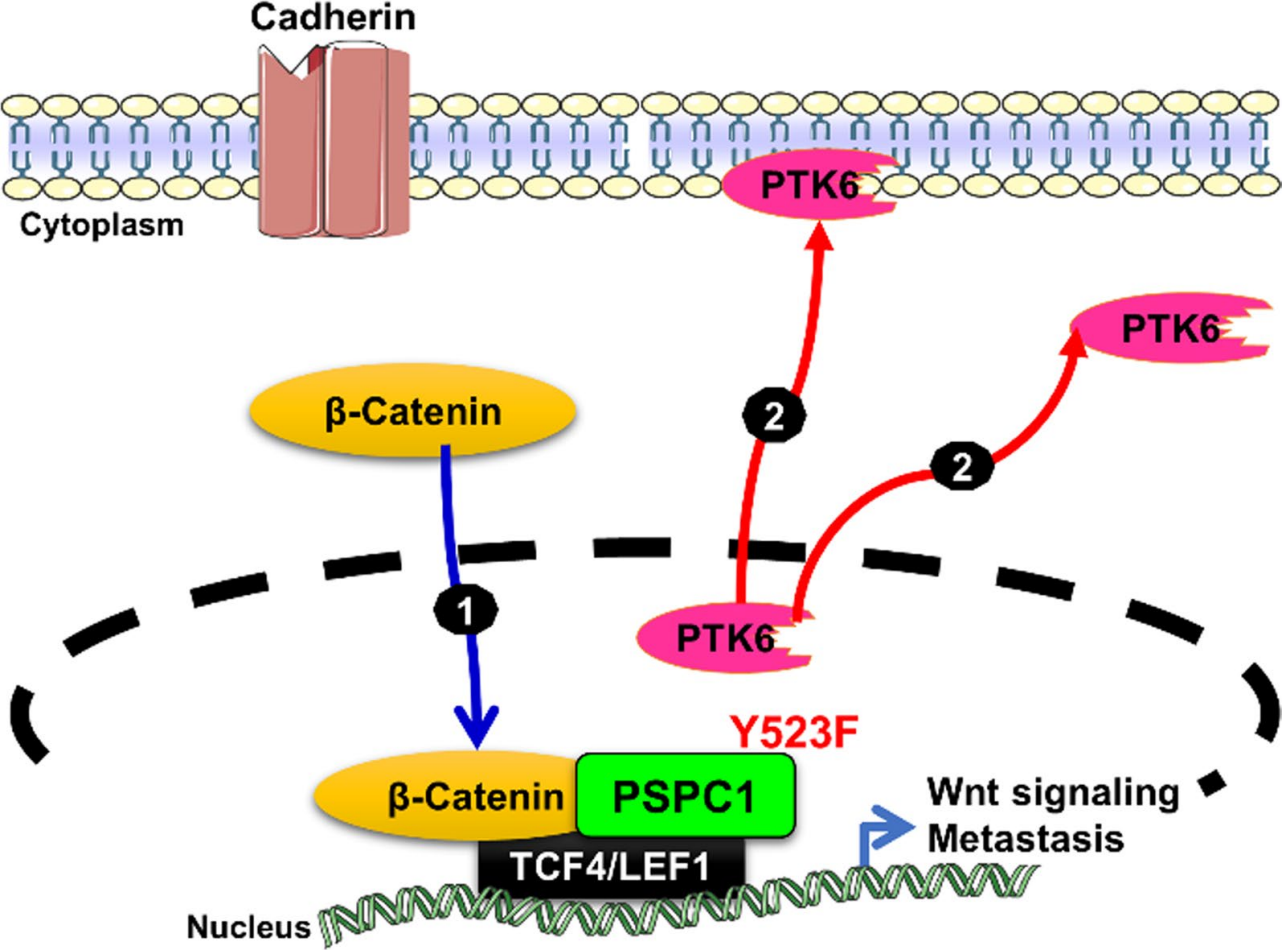
PSPC1 is the contextual determinant of PTK6/β-catenin reciprocal nucleocytoplasmic shuttling. (1) In cells of advanced cancer with high PSPC1 expression, PSPC1 upregulation or mutation of amino acid residue 523 in PSPC1 from tyrosine to phenylalanine (PSPC1-Y523F) can determine oncogenic subcellular translocation to exert synergistic effects on the translocation of cytoplasmic β–catenin to the nucleus and preferentially interact with PSPC1 to facilitate synergistic oncogenic effects (2) PTK6 can translocate to the cytoplasm and cell membrane as an oncogene to facilitate synergistic oncogenic effects such as epithelial-to-mesenchymal transition (EMT), Wnt3a autocrine signaling and stemness promoting metastasis

PTK6 was identified via its interaction with PSPC1 in the nucleus by proteomic analysis and acts as a tumor suppressor to abrogate the oncogenic effects of PSPC1. Indeed, in premalignant cancer cells with low PSPC1 expression, PTK6 is sequestered by its tyrosine-phosphorylated substrate and interacts with PSPC1-phospho-Y523 in the nucleus to suppress the tumorigenic functions of PSPC1. A newly induced antibody against Y523-phosphorylated PSPC1 was applied in immunohistochemical (IHC) experiments on human HCC tissues, which demonstrated that lower expression of Y523-phosphorylated PSPC1 was associated with poor survival in HCC patients. Diminished expression of Y523-phosphorylated PSPC1 indicated loss of PTK6 sequestration in the nucleus and shuttling to the cytoplasm and cell membrane to activate oncogenic PTK6. However, in cells of advanced HCC with high PSPC1 expression, PTK6 can translocate to the cytoplasm and cell membrane as an oncogene, and reciprocally, cytoplasmic β–catenin can translocate to the nucleus to interact with PSPC1 to facilitate synergistic oncogenic effects such as EMT, Wnt3a autocrine signaling and stemness toward metastasis. Therefore, PSPC1 is the contextual determinant for the reciprocal oncogenic subcellular translocation of cytoplasmic PTK6 and nuclear β-catenin to exert synergistic effects on oncogenic tumor progression (Fig. 2) [27]. We found that PSPC1 overexpression can also be a contextual activator to stimulate the nuclear translocation of β-catenin with enhanced interaction of PSPC1 and β-catenin in the nucleus and to upregulate Wnt3a autocrine signaling to potentiate synergistic oncogenic progression mediated by PSPC1 and PTK6 in HCC cells. Moreover, targeting the PSPC1/PTK6 interaction with PSPC1 C-terminal 131 polypeptide (PSPC1-CT131) as an innovative therapeutic agent in HCC cells was found to reverse the nucleocytoplasmic shuttling of PTK6 and β-catenin, suppress the expression of the autocrine oncogenic growth factors Wnt3a and TGF-β, and inhibit tumor progression and metastasis in vivo in HCC mouse models.

## TGIF1 is the contextual determinant of PML nucleocytoplasmic shuttling

**Fig. 3:**
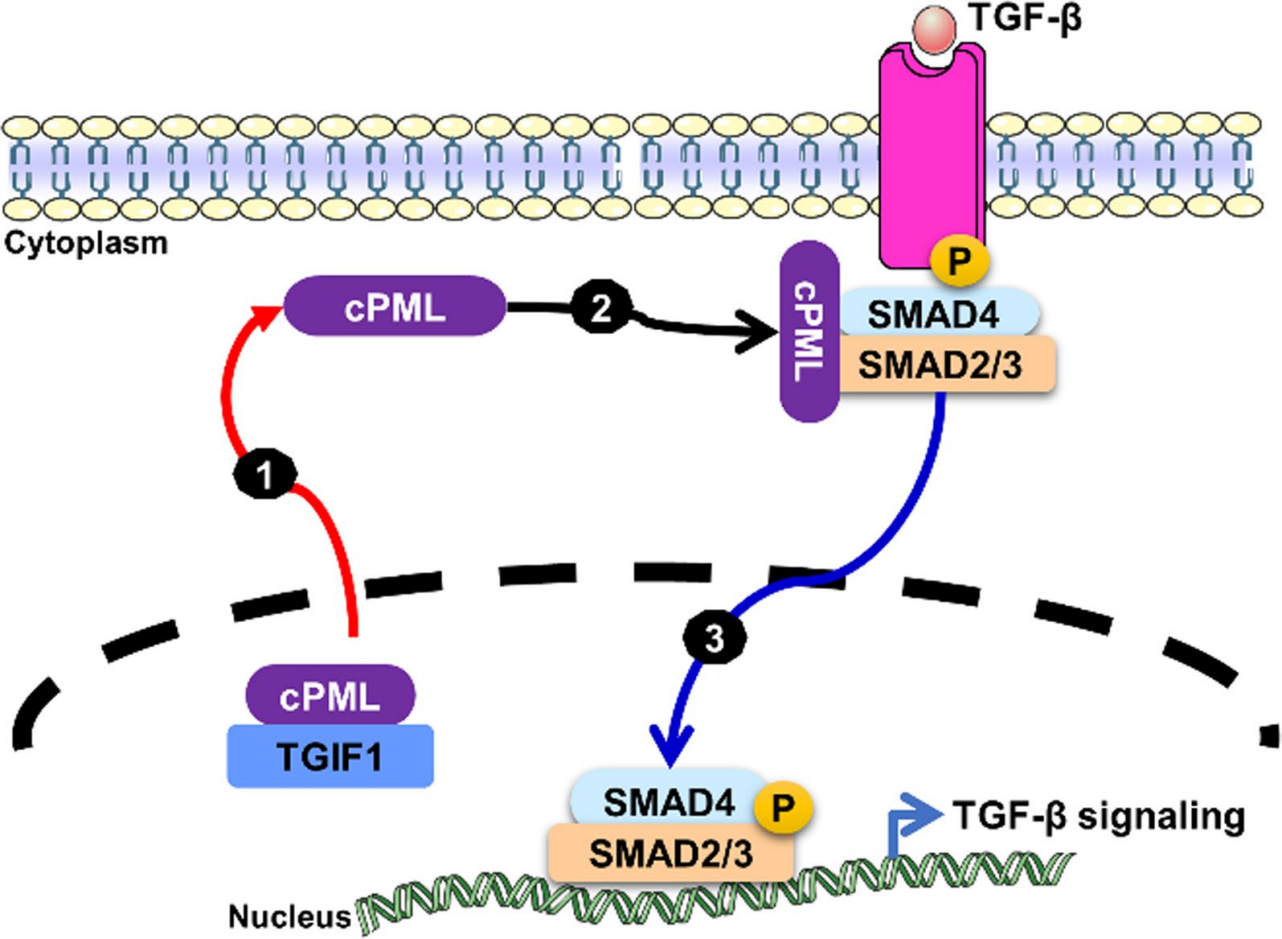
TGIF1 is the contextual determinant of PML nucleocytoplasmic shuttling. (1) PML delocalization was initiated by escape of nuclear interaction with TGF-β Induced Factor Homeobox 1 (TGIF1) to prevent cPML translocation to the cytoplasm. (2) Cytoplasmic promyelocytic leukemia (cPML) appears to preferentially interact with phosphorylated Smad2/3 and acts as an essential activator of TGF-β signaling (3) Activation of the canonical TGF-β signaling pathway

TGF-β Induced Factor Homeobox 1 (TGIF1) is known to function as a nuclear corepressor of TGF-β signaling through multiple mechanisms, such as recruiting Smad2/4 and histone deacetylases (HDACs) to form a transcriptional repressor complex on the Smad target promoter to repress the transcription of TGF-β signaling [49], interacting with the E3 ubiquitin ligase Tiul1 (TGIF-interacting ubiquitin ligase 1, or WWP1, WW Domain Containing E3 Ubiquitin Protein Ligase 1) to degrade Smad2 via ubiquitin-dependent proteasomal degradation [50], and sequestering cytoplasmic promyelocytic leukemia (cPML) in the nucleus to prevent its shuttling to the cytoplasm to interact with Smad2/3 and TGF-β receptors (TβRI and TβRII) to activate oncogenic TGF-β signaling in cancer cells [29, 30, 51] (Fig. 3).

Promyelocytic leukemia protein (PML) was originally found in patients with a rare subtype of acute myeloid leukemia (AML) called acute promyelocytic leukemia (APL), which expresses a PML–RARα fusion oncoprotein encoded by *PML* and the retinoic acid receptor alpha (RARα) chromosomal translocation between chromosomes 15 and 17, t(15;17) [52–55]. In normal cells, nuclear PML forms nuclear multiprotein complexes called PML nuclear bodies (NBs) and functions as a tumor suppressor to modulate the transcription of p53 and Rb, leading to modulation of apoptosis, cell proliferation, and cell senescence [56]. However, some PML isoforms derived from alternative splicing of mRNAs without nuclear localization signal (NLS) motifs, called cytoplasmic PML (cPML) isoforms, were identified in the cytoplasm and demonstrated to activate TGF-β signaling mediating tumor suppression, apoptosis and senescence in a physiological context [29]. On the other hand, cPML was also shown to interact with XPO1 and to be exported from the nucleus to the cytoplasm to promote association with the Smad2/3-dependent canonical TGF-β signaling pathway to enhance EMT and invasion of prostate cancer cells [30] (Fig. 3). Clinically, treatment of APL patients and other cancer cells with arsenic trioxide (ATO) and/or all-trans-retinoic acid (ATRA) was shown to cause proteasomal degradation of PML–RARα and cPML to prevent their nucleocytoplasmic shuttling and reverse their tumorigenic effects [57–59].

## NPM1 is the contextual determinant of NPM1 and HEXIM1 nucleocytoplasmic shuttling

NPM1, encoded a protein called nucleophosmin or nucleolar phosphoprotein B23, is associated with nucleolar ribosome biogenesis, nucleolar protein transportation, formation of G-quadruplex nucleic acids, modulation of histone chaperone and chromatin remodeling, and promotion of cell proliferation via the Myc-ARF-p53 axis and SUMOylation [60]. NPM1 is a nucleolar nucleocytoplasmic shuttling protein that has been shown to interact with different protein partners in response to different cellular functions and even redox stress. Overexpressed NPM1 can translocate to the nucleolus via nucleocytoplasmic shuttling to sequester HDM2 and disrupt the p53–HDM2 interaction to promote DNA damage and viral stress-induced activation of p53 [61]. Additionally, NPM1 nucleolar translocation is crucial for c-Myc nucleolar localization and c-Myc-mediated transcription to regulate rRNA synthesis to induce fibroblast proliferation and transformation [62].

NPM1 is commonly upregulated, mutated and chromosomally translocated in hematopoietic cancers and solid tumors [63]. In acute myelogenous leukemia (AML), NPM mutants are commonly shown to lack a folded C-terminal domain (NPM1c+) and are expressed in the cytoplasm in association with AML development in patients [64]. Therefore, NPM1c + mutations serve as a biomarker for first-line screening in AML patients with myeloid neoplasms defined by the WHO classification. Interestingly, it has been shown that NPM1c + can drive TGF-β/Smad signaling via the cytoplasmic form of the promyelocytic leukemia (cPML) protein to contribute to EMT and invasion during the pathogenesis of NPM1c + AML [65].

**Fig. 4:**
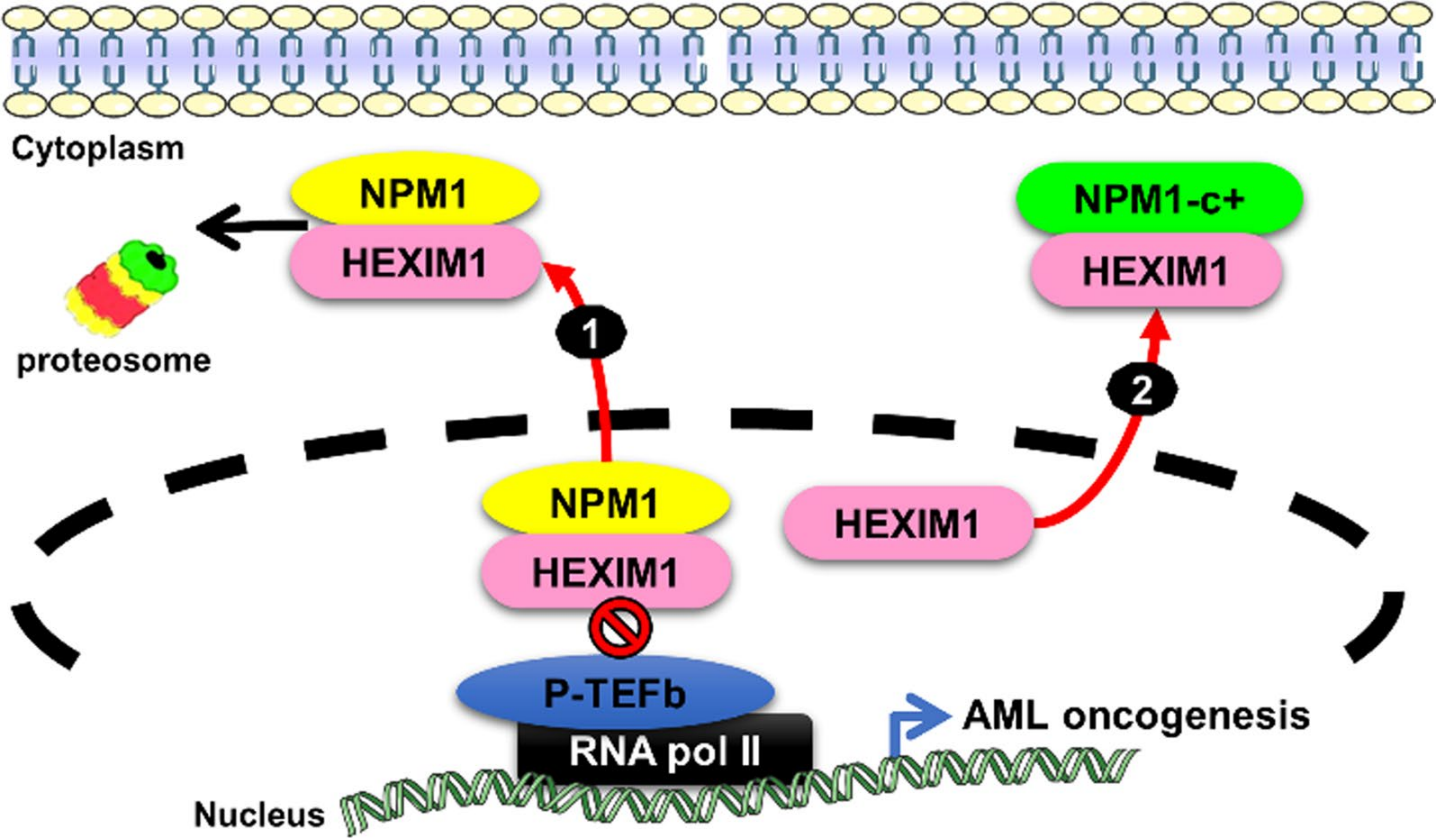
NPM1 is the contextual determinant of NPM1 and HEXIM1 nucleocytoplasmic shuttling. (1) Nucleophosmin (NPM1) binds to HEXIM1 leading to proteasome-mediated degradation of HEXIM1 and resulting in activation of P-TEFb-dependent RNA Pol II transcription. (2) The cytoplasmic mutant of NPM1 (NPM1c+) sequesters HEXIM1, an inhibitor of P-TEFb, in the cytoplasm, thereby resulting in a higher level of RNA Pol II transcription in acute myeloid leukemia (AML)

Previous studies have also demonstrated that NPM1 can interact with hexamethylene bisacetamide-inducible protein 1 (HEXIM1), an inhibitor of positive transcription elongation factor b (P-TEFb) and a novel positive regulator of p53. In NPM1c + AML cells, NPM1c + sequesters HEXIM1 in the cytoplasm, resulting in a high level of RNA Pol II transcription (Fig. 4) [32]. By exploiting the interacting domain of the basic region (BR) of HEXIM1, which mediates the binding with NPM1 that is responsible for ubiquitination by the human double minute 2 protein to promote degradation, an innovative cytotoxic peptide fused with a cell-penetrating or breast cancer-specific peptide—the HEXIM1 BR peptide—was developed and found to trigger rapid specific cytotoxic killing of breast cancer cells [31]. In summary, elucidating how NPM1 abnormalities and/or aberrant nucleocytoplasmic shuttling contributes to tumor progression is emerging as a need for developing innovative targeting strategies for the improvement of cancer therapy.

## Mortalin is the contextual determinant of p53 nucleocytoplasmic shuttling

In normal cells, p53 is expressed in such a low quantity that it is undetectable by immunofluorescence and immunohistochemistry but is critical for modulating cell cycle progression, apoptosis, DNA repair and genomic stability as the guardian of the genome to maintain cell homeostasis [66]. In cancer or stressed cells, p53 is classified as a tumor suppressor and acts as a sensor of multiple forms of cellular stresses; it has been known for decades to have different subcellular localizations (in the nucleus and cytoplasm), but both the detailed mechanism responsible for its retention and its potential clinical application remain obscure [67, 68].

Mortalin is a 74 kDa mitochondrial chaperone protein that is a member of the heat shock protein 70 (Hsp70) family [69]. Mortalin has been demonstrated to exhibit different subcellular distributions in normal and cancer cells and performs multiple functions in processes ranging from stress responses and intracellular trafficking to cell proliferation and tumorigenesis [70]. The molecular functions of mortalin are commonly determined by its interaction partners and subcellular localization [71]. Mortalin is the contextual determinant of cytoplasmic sequestration of p53, as demonstrated by coimmunoprecipitation experiments in colon cancer cells (in which it was localized in puncta) and by immunohistochemistry in colon cancer tissues [72]. Loss of the tumor-suppressive functions of nuclear p53 by its cytoplasmic sequestration is a general mechanism for p53 inactivation leading to loss of the capability to modulate centrosome duplication and genome stability in undifferentiated neuroblastoma and other cancers [67, 73, 74]. Indeed, mortalin overexpression has been identified as a biomarker of HCC metastasis and recurrence [75].

**Fig. 5:**
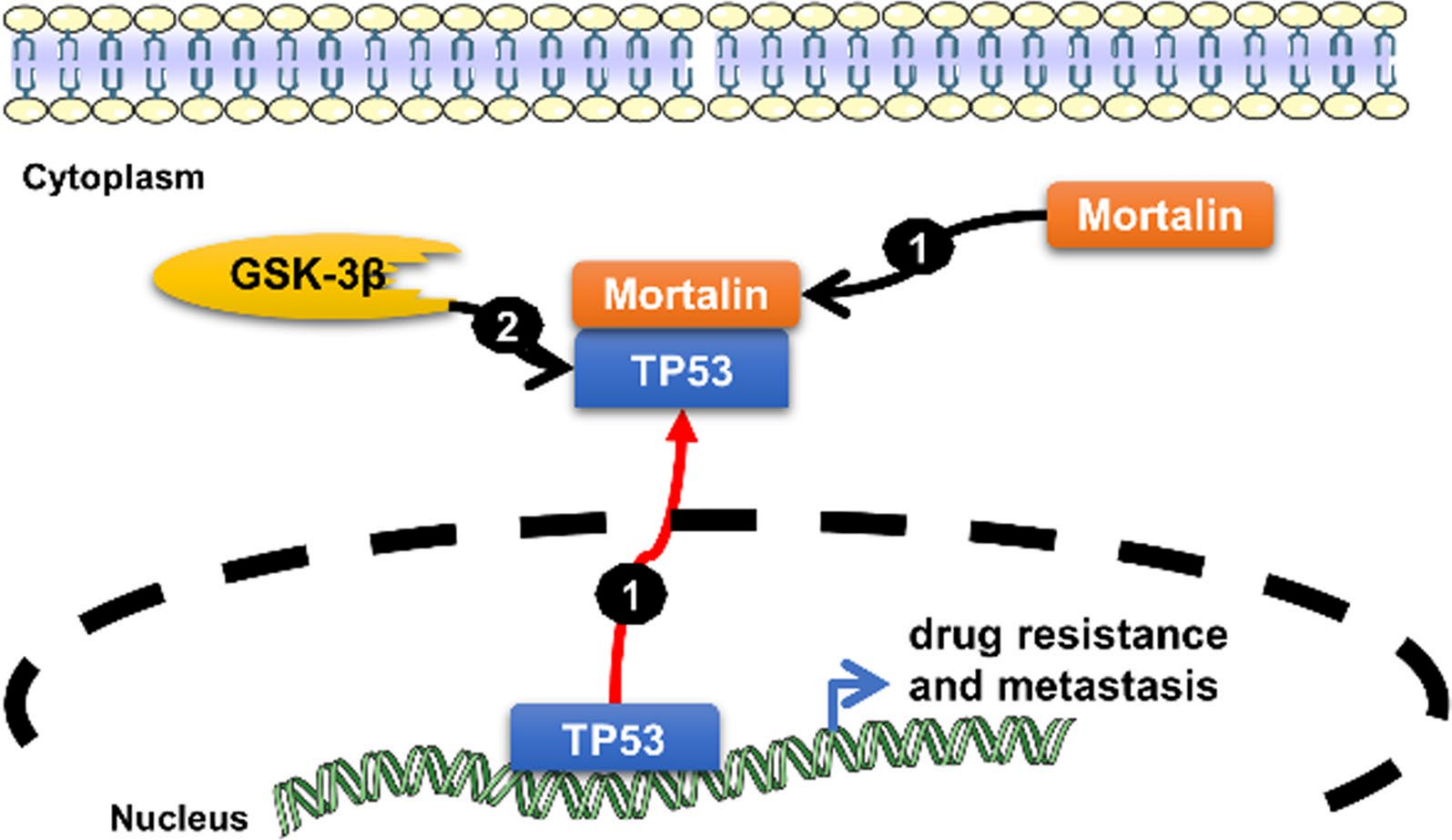
Mortalin is the contextual determinant of p53 (TP53) nucleocytoplasmic shuttling. (1) Cytoplasmic p53 is sequestered by the heat shock protein mortalin in cancer cells through multiple mechanisms, including excess nuclear export of p53, defective cytoplasmic degradation, retention by cytoskeletal proteins and other potential mechanisms. (2) Glycogen synthase kinase-3 beta (GSK-3beta) phosphorylates p53 to induce its cytoplasmic localization, thereby enhancing constitutive cytoplasmic localization of p53 under ER stress to prevent p53 stabilization and p53-mediated apoptosis upon DNA damage [76]

After molecular dissection of the mortalin-p53 interaction, mortalin was found to bind to the p53 C-terminal tetramerization (TET) domain (323 ~ 355 amino acids) in cancer cells but not in normal cells, and this observation was applied to develop inhibitors targeting cytoplasmically sequestered p53-mortalin and reactivate the tumor-suppressive activities of p53 via shuttling it back to the nucleus. At least three strategies to disrupt the cytoplasmic sequestrated mortalin-p53 interaction, including (a) a cytoplasmically localized p53 polypeptide containing the C-terminal residues 323–337 as a competitor of p53 in the p53-mortalin interaction [34], (b) a cationic rhodacyanine dye analog MKT-077 bound to mortalin [35], and (c) shRNA-mediated mortalin silencing [33], were shown to release p53 from mortalin–p53 complexes, to mediate the nuclear translocation and reactivation of tumor-suppressive p53, and to cause growth arrest and apoptosis in different human liver cancer cells (Fig. 5).

## EBP50 is a contextual determinant of EBP50 and β-catenin nucleocytoplasmic shuttling

Dysregulation of the Wnt/β-Catenin signaling pathway plays important roles in cancer stemness and tumor progression [77]. After activation with Wnt ligands, unphosphorylated and stabilized β-catenin enters the nucleus through nucleocytoplasmic shuttling and accumulates in the nucleus, where it forms a transcription complex with the transcription factor T cell factor (TCF) to transactivate downstream oncogenic gene expression [78].

**Fig. 6:**
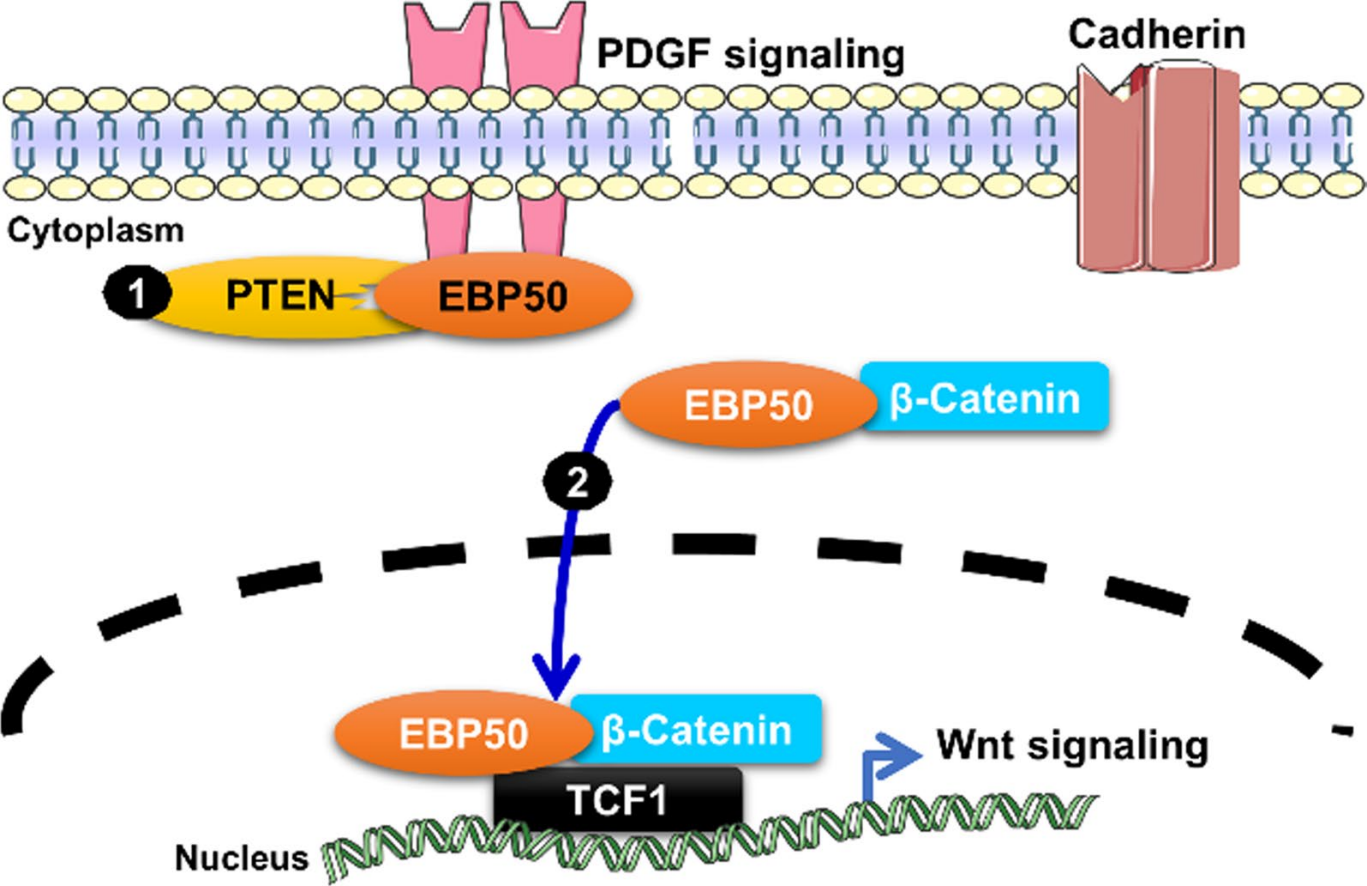
EBP50 is a contextual determinant of EBP50 and β-catenin nucleocytoplasmic shuttling. (1) EBP50 interacts with the PTEN tumor suppressor to attenuate PDGF receptor signaling. (2) During tumor progression, EBP50 translocates to the nucleus to stabilize β-catenin/TCF-1 or dnTCF-1 for activation of Wnt oncogenic signaling

ERM-binding phosphoprotein 50 (EBP50) (also called SLC9A3 regulator or NHERF1, Na+/H + exchanger regulatory factor 1), containing two tandem PDZ domains and a C-terminal ERM–binding (ezrin-radixin-moesin-binding) domain, was reported to be an adaptor for protein interactions in multiple subcellular localizations with divergent functions [79]. EBP50 is a known β-catenin-associated protein and is overexpressed in the nucleus of HCC cells to stabilize and enhance the oncogenic transcriptional activity of β-catenin to promote tumor growth (Fig. 6) [80]. In contrast, EBP50 also acts as a tumor suppressor by recruiting PTEN to PDGFR at the plasma membrane to form the EBP50/PTEN/PDGFR ternary complex to suppress PI3K activation in normal cells [36]. Nuclear EBP50 was shown by chromatin immunoprecipitation assays to occupy consensus DNA sequences in Wnt-responsive binding motifs, and it stabilizes β-catenin/TCF-1 complexes or even β-catenin/dnTCF-1 complexes (dnTCF-1 is a truncation mutant of TCF-1 lacking the β-catenin-binding domain and acting as a transcriptional suppressor) to form a ternary molecular complex to enhance Wnt/β-catenin signaling and downstream c-Myc and cyclin D1 oncogene expression. Moreover, the expression of nuclear EBP50 in human colorectal carcinoma cell lines was shown to enhance cell cycle progression, anchorage-independent growth, and tumorigenesis in a mouse model and was found to be associated with poor clinical outcome in patients with human primary colorectal tumors [37]. Knockdown of EBP50 with siRNA (siEBP50) disrupted the β-catenin and TCF-1 interaction to downregulate downstream c-Myc and cyclin D1 expression, suppress colon cancer cell proliferation and colony formation, and inhibit tumor growth in a NOD-SCID mouse model.

## Conclusions

Aberrant nucleocytoplasmic shuttling is emerging as a hallmark in cancer progression owing to alterations in diverse mechanisms in the protein shuttling machinery that are commonly reported in multiple cancer types. Although emerging therapeutic agents targeting consensus determinants, such as SINE inhibitors targeting XPO1, have gained accelerated FDA approval and been evaluated as anticancer therapies in multiple phase I/II clinical trials in the last 3 years, endeavors to better understand the molecular mechanisms and select other factors in nucleocytoplasmic shuttling for the development of innovative cancer therapies could be promising. Here, we suggest that PSPC1, TGIF1, NPM, Mortalin and EBP50 are contextual determinants that activate nucleocytoplasmic shuttling of tumor suppressors and oncogenes with opposite tumorigenic effects in different subcellular locations. Therefore, the critical nucleocytoplasmic shuttling determinants should be potential targets of developing therapeutic agents to inhibit aberrant determinants and shuttling proteins as an innovative anticancer approach leading to synergistic tumor suppression.

## Data Availability

Not applicable.
